# Age-Related Modifications to the Magnitude and Periodicity of Neuromuscular Noise

**DOI:** 10.1371/journal.pone.0082791

**Published:** 2013-12-12

**Authors:** Navrag B. Singh, Niklas König, Adamantios Arampatzis, William R. Taylor

**Affiliations:** 1 Institute for Biomechanics, Department of Health Science and Technology, Eidgenössische Technische Hochschule Zürich, Zürich, Switzerland; 2 Department of Training & Movement Science, Humboldt-Universität zu Berlin, Berlin, Germany; University of Sydney, Australia

## Abstract

**Background:**

Evaluation of task related outcomes within geriatric and fall-prone populations is essential not only for identification of neuromuscular deficits, but also for effective implementation of fall prevention strategies. As most tasks and activities of daily living are performed at submaximal force levels, restoration of muscle strength often does not produce the expected benefit in functional capacity. However, it is known that muscular control plays a key role in the performance of functional tasks, but it remains unclear to what degree muscular control and the associated neuromuscular noise (NmN) is age-related, particularly in the lower-extremities.

**Objectives:**

The aim of this study was to determine the effects of age and fall-pathology on the magnitude as well as the frequency of NmN during lower extremity force production.

**Methods:**

Sixteen young healthy adults, as well as seventy elderly women (36 healthy, 34 elderly fallers), performed force production tests at moderate levels (15% of maximum voluntary isometric contractions).

**Results:**

Elderly fallers exhibited the highest magnitude of NmN, while the highest frequency components of NmN tended to occur in the healthy elderly. Young subjects exhibited significantly more power in the low frequency ranges than either of the elderly groups, and had the lowest levels of NmN.

**Conclusion:**

These data suggest increased degeneration of muscular control through greater NmN in elderly fallers compared to healthy elderly or young subjects. This could possibly be associated with muscle atrophy and lower levels of motor unit synchronisation.

## Introduction

Age related modifications to the neuromuscular system [1] include muscular atrophy and a deterioration of muscular function [2], resulting in a prevalence towards falls [3] and injury, with fatality as a frequent consequence [4], [5]. The evaluation of task related outcomes within geriatric and fall-prone populations is therefore essential for understanding motor related deficits, but also for effective implementation of fall prevention strategies.

Muscle atrophy is an important intrinsic characteristic that severely restricts independent living among the elderly [6]. The quantification of muscle strength in the upper (e.g. grip strength) as well as the lower extremities (e.g. knee flexion and extension) has therefore become a key metric for evaluating task performance in clinical settings [6]. However, it can also be argued that rehabilitation programs focusing on improvements in muscular strength have not achieved the expected benefits in functional performance [7]. One possible reason for such a discrepancy could arise from the fact that most activities and motor tasks are performed at submaximal levels and therefore a focus on e.g. muscular control and coordination, rather than muscle strength itself, might be more valid targets for improving functional task performance.

During submaximal contractions, muscles are unable to generate purely constant forces [8]–[10], but instead, the output fluctuates around the desired force level. As a result, the quality of the force produced can be assessed by quantifying both its accuracy, as the difference between a target force and the actual force generated [11], as well as the magnitude of the fluctuations, which can be considered a measure of steadiness [8]. Furthermore, the periodicity (or spectral content) of the output signal can provide an insight into the rhythmic modulation of multiple motor units [11], [12]. Studies conducted primarily on the upper-extremity muscles have revealed an association between force fluctuations and; the recruitment patterns of muscle motor units (MMUs) [8], [9], [13], [14], and the variability in the discharge rate of action potentials [14]–[19]. As a result of these characteristics, the fluctuations observed during static isometric force production provide a quantification of *Neuromuscular noise* (NmN) [20]. Since the motor unit pool is susceptible to apoptosis during the natural course of ageing and associated muscle atrophy [2], it seems plausible that changes in the structure of force output will be also be observed with progressive aging and/or neuromotor pathology.

Characterisation of NmN has allowed an in-depth understanding of the ageing motor system in the upper extremities [8], [12], [21]. Similar investigations in the lower-extremities, however, have generally ignored the impact of NmN on task performance [8], [10], despite the fact that: 1) the type and properties of muscles in the lower extremities are different from those in the upper extremities [8], and 2) most activities of daily living (e.g. walking) require predominantly muscles in the lower-extremity. Recent studies have shown that NmN [22] affects whole-body task performance, but it remains unknown whether, and to what degree, NmN is age-related and how it may affect the risk of falling. While strength and balance related discrepancies have been well established as risk factors and clinical biomarkers for falling [3]–[5], a complete characterisation and understanding of NmN in the sensorimotor system among elderly fallers remains elusive [3]–[5], [22].

In an attempt to better understand the age- and fall-related modifications involved during force production, the aim of this study was to evaluate the magnitude as well as the periodicity of NmN during lower extremity force production in young vs. elderly individuals. We hypothesized that an increase in magnitude as well as a change in the spectral structure of the NmN will be observed in elderly adults compared to young healthy individuals. In addition, our secondary hypothesis was that an increase in magnitude and a change in the spectral structure of NmN will be observed in elderly subjects that have experienced a fall in the previous 12 months. By incorporating a cohort of elderly healthy as well as faller cohorts the vision of the study was to characterise and understand the degeneration of NmN with motor related deficits and thereby enable the assessment of neuromuscular deficits in clinical settings.

## Methods

### Ethics Statement

This research was conducted according to the principles expressed in the Declaration of Helsinki. All participants provided written informed consent before beginning the experimental procedures, which were approved by the Ethics Committee at the Charité University Hospital Berlin.

### Participants

Sixteen young healthy adults (8 males and 8 females) from the local community, with no self-reported injuries, illnesses, or musculoskeletal disorders volunteered to participate in this study. Their mean (SD) age, body mass, and height were 28.9 (2.3) years, 71.8 (13.3) kg, and 176.8 (11.4) cm respectively.

In addition, seventy elderly women (34 who had experienced at least 1 fall within the previous 12 months - ‘‘elderly faller’’; 36 healthy controls – ‘‘healthy elderly’’) who completed the entire experimental protocol were included in this study. Both groups were homogenous in terms of age, weight and height with a mean (SD) of: 69.8 (4.8) years, 69.7 (10.2) kg and 163.1 (6.6) cm for elderly fallers, and 69.2 (4.6) years, 67.7 (10.7) kg and 162.1 (6.0) cm for healthy elderly cohorts respectively.

### Experimental Design and Procedures

Force fluctuations were considered a measure of NmN and were assessed in the knee extensors. While a full description of the methods is available in the literature [11], a brief overview is provided here: Participants were seated in a standardised position on a dynamometer (Biodex Medical Systems Inc., USA). Before each measurement, the flexion/extension rotation axis of the knee was aligned with the rotational axis of the dynamometer. Knee extension measurements were then conducted in a seated position with 90 degrees of flexion at the knee. Prior to the start of each force fluctuation session, maximum voluntary isometric contractions (MVICs) were obtained by providing standardised instructions and verbal encouragement, with subjects aiming to reach peak exertion 2–3s after the start of the trial. MVICs, which lasted for 5s, were measured three times with a minimum of 30s pause between contractions [20]. The peak value of the three contractions was then used as the respective MVIC.

Objective or target torque levels were provided visually as constant plots, displayed as a horizontal line on a monitor placed in front of the participant. The level of torque was set at 15% MVIC, overlaid by the actual knee extensor torque produced (real-time visual feedback at 10 Hz) for a duration of 15s. After undertaking familiarising practice sessions, all tests were conducted a minimum of three times.

### Data Analysis

All torque measurements were collected using Labview (Labview 8.6, National Instruments, Inc., USA). From each trial, the first 5 and the last 2 seconds of torque output were removed to avoid transients during initiation or termination of the trials. All data were then low pass filtered (4^th^ order, zero-phase lag, Butterworth filter, 25 Hz cut-off frequency).

In order to assess the quality of force output, the normalized mean absolute error (NMAE) and normalized standard deviation of absolute error (NSAE) were evaluated [11]. In order to evaluate these parameters the error between the actual force produced and the target signal (15% MVIC) were first rectified. The NMAE and NSAE were then obtained as the ratio of mean rectified error and standard deviation of rectified error to the individual’s MVIC respectively. In addition, the coefficient of variation (CoV) of the produced extensor torque represented the NmN and was evaluated as the ratio of the standard deviation to the mean of the force production signal. In order to assess the periodicity of NmN, the mean power frequency (MnPF) of the force production signal was evaluated, as well as the power in the spectrum (normalised to total power), which was divided into two bands of known physiological importance [11]: low (0 – 4 Hz; NPL) and high (8 – 20 Hz; NPH) frequencies.

### Statistical Analysis

The levels as well as the spectral content of NmN were compared between the young and the elderly subjects. Here, a one-way mixed-factor ANOVA with 3 levels (*young, healthy elderly, elderly fallers*), was conducted to analyse the effect of age and fall-related modifications on measures of NmN. Least significant differences (LSDs) were used to illustrate post-hoc comparisons. A significance level of p < 0.05 was set for all analyses. The SPSS software package (v20 SPSS Inc., USA) was used for all statistical analyses.

## Results

**Figure 1:**
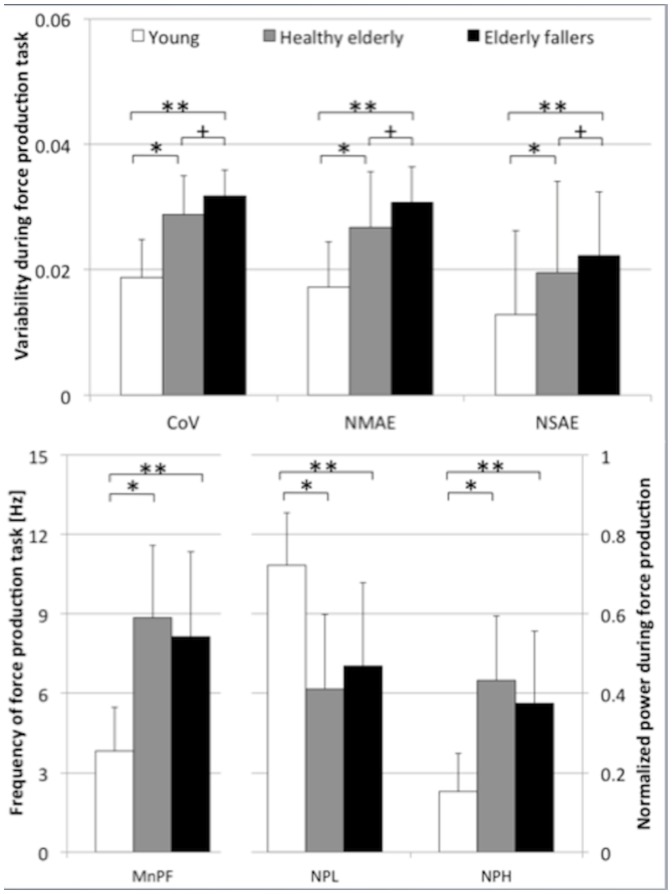
Age-related changes in the magnitude and periodicity of neuromuscular noise. Effects of age (young adults; white bars, vs. healthy elderly; grey bars) as well as fall related differences (healthy elderly vs. elderly fallers; black bars) on the magnitude of neuromuscular noise (NmN; Top), namely CoV signal, normalized mean (NMAE) and standard deviation of absolute error (NSAE), during force production at 15% MVIC. The measures of periodicity in NmN (bottom), namely mean power frequency (MnPF), normalized power in the low, 0 – 4 Hz (NPL) and high (8 – 20 Hz; NPH) frequency bands for young adults, healthy elderly and elderly fallers are also shown. * represents significant differences at p < 0.05 between the healthy young and healthy elderly, ** represents significant differences at p < 0.05 between the healthy young and elderly fallers, whereas + represents significant differences at p < 0.05 between healthy elderly and elderly fallers.

The magnitudes of CoV, NMAE as well as NSAE were consistently the lowest in the young adults, followed by the healthy elderly and finally greatest in the elderly fallers (Figure 1; Top). The extent of NmN was approximately 50% higher in the healthy elderly as compared to the young adults (p < 0.05) and 10% higher in elderly fallers compared to their healthy counterparts (p < 0.05).

The force produced by the healthy elderly (MnPF  =  8.9 Hz) also fluctuated at a higher frequency than the young adults (MnPF  =  3.8 Hz; p < 0.05; Figure 1 - bottom left). Approximately 40% of the force fluctuated at high frequencies in the healthy elderly (visible in the 8–20 Hz band; Figure 1 - bottom right), compared to the young subjects, who performed the same task with less than 20% fluctuations occurring at high frequency (p < 0.05). Although not significant, the elderly fallers displayed a tendency towards force production at lower frequencies, demonstrated by the increased power in the 0–4 Hz band (p = 0.128) and less power at higher frequencies (8–20 Hz band; p = 0.084), than the healthy elderly subjects.

## Discussion

As most tasks and activities of daily living are performed at submaximal force levels, predominantly requiring activation of lower-extremity muscles, this study targeted an understanding of the modifications that occur in neuromuscular noise between young and elderly adults, as well as in elderly subjects with a fall history, during moderate (15%) force production tasks. The results of this study revealed that the largest levels of NmN occurred in the elderly fallers, followed by the healthy elderly and the healthy young cohorts. Surprisingly, however, we found that the healthy elderly group displayed a tendency towards possessing the highest mean fluctuation frequencies during the force production task among the three groups (Figure 1, bottom). As a result, it could be considered that the neuromuscular noise also occurred at the highest mean frequencies in this group, even though it had a lower magnitude (Figure 1, top) than in elderly fallers. In the case of elderly fallers, however, NmN seemed to occur predominantly at low frequencies (in the 0–4 Hz, not significant), even though this frequency was still well above that observed in young healthy adults (p<0.05). During force production, recently recruited MMUs create a fluctuation in the force output. After a perturbation such as fatigue [8], [9], [11], [23], [24], the MMUs exhibit a tendency towards more synchronized firing, commonly known as MMU synchronization [8], [12], as a compensation strategy to allow continued force production [25]. This phenomenon results in an additional higher frequency component in the force output (seen in the 8–20 Hz frequency band), generally acknowledged as *physiological tremor* [13], [17]. Our results have revealed a similar modification to the structure of the force production signal in our elderly cohorts (Figure 1, bottom), probably caused by higher levels of MMU synchronisation in these subjects [12], [19]. Interestingly, although not significant, elderly fallers did exhibit a tendency towards reduced power in this high frequency tremor 8–20 Hz region (p = 0.084), suggesting reduced levels of MMU synchronization probably due to a reduction in the number of MMUs available [2]. Here, it seems likely that additional muscle atrophy diminishes their ability to use MMU synchronisation as a compensation strategy, and results in superimposed action potentials [26], hence the increased magnitude of NmN but at lower frequencies.

The measures of NmN considered in this study were CoV, NMAE and NSAE. The CoV was the ratio of the standard deviation to the mean of the actual force produced by each individual, whereas both the normalized mean and normalized standard deviation of the absolute error quantify the inaccuracy and the unsteadiness during force production. The CoV_,_ on the other hand, quantifies the variation (NmN) in terms of the force produced by the individual and is therefore likely to be constant across different levels of force produced despite the known signal dependence of NmN within an individual [27].

**Figure 2:**
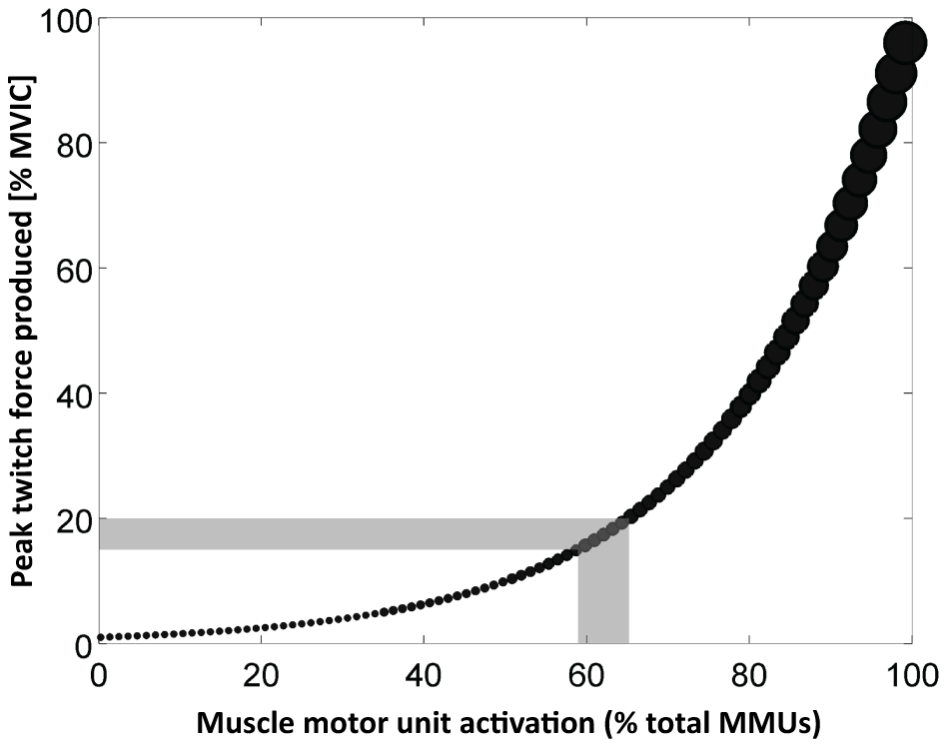
Relationship between neuromuscular noise and activation of muscle motor units. This schematic (adapted from Fuglevand and co-workers[14]) demonstrates the mechanisms of force production (y-axis) as a function of activation of muscle motor units (MMUs; x-axis). The recruitment or activation of the MMUs is predominantly guided by the size principle [28], with the smallest units recruited first. The figure demonstrates that recruitment of approximately 60 – 68% of the existing MMUs is required for force production at 15 – 20% of maximum capacity (grey region, [14]). Due to muscle atrophy in elderly individuals, the motor unit pool needs to recruit larger MMUs compared to healthy young adults in order to produce the same levels of force output. These mechanisms provide a plausible explanation for the larger levels of NmN observed during the force production task in the elderly subjects.

The levels of NmN in healthy elderly individuals reported here were similar to those reported elsewhere among fatigued healthy young adults [11]. The similarities between the extent of NmN in elderly individuals and fatigued young adults occur due to the previously established physiological MMU recruitment mechanisms that allow force production [8], [14]. During submaximal isometric force production, MMUs are recruited according to the size principle [28], with the smallest recruited first and subsequently the larger MMUs. The distribution of these MMUs is thought to be driven empirically with the following relationship [14]:
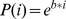



where *P(i)* is the peak twitch force of the *i* ^th^ MMU. During the production of about 15% of the maximum force, approximately 60% of the MMUs within the entire pool will be recruited [14] (Figure 2).

When a muscle is fatigued, the MMUs are unable to produce a desired force [29], and eventually fresh and larger MMUs are recruited to produce the required output [28], [29]. These physiological changes, including increased synchronisation, lead to an increase not only in the magnitude of NmN [11], [23], [24], [30], but also in the frequencies of fluctuation during force production [11], [24]. In elderly adults, the degeneration of MMUs associated with muscle atrophy, leads to remodelling of the entire pool [2]. The loss of MMUs in the elderly may cause the existing MMUs to re-innervate the remaining muscle fibres [2], possibly explaining the higher levels of NmN, as well as the larger periodicity in force production that has been reported in this study among the healthy elderly cohort. Interestingly, this observation is consistent with the increase in NmN seen in young healthy subjects after fatigue [11], [24], although the underlying mechanisms remain to be investigated.

In this study, elderly fallers possessed larger levels of NmN compared to the healthy elderly. However, trends suggest that this increase in the magnitude of noise did not occur at higher frequencies (cf. Figure 1, bottom; MnPF as well as NPH). These data suggest that fallers might possess a limited capacity to compensate for the loss of MMUs. It is likely that muscle atrophy in elderly fallers limited the ability to adopt synchronisation strategies during force production, but confirmation of these results is required in studies using larger cohorts. This study therefore provides a basis for understanding the mechanisms underlying muscular force production and the associated neuromuscular noise among healthy adults, as well as those that might suffer from motor-related pathologies.
